# Psilocybin-assisted Existential, Attachment and RelationaL (PEARL) therapy for patients with advanced cancer: protocol for a multi-method feasibility trial

**DOI:** 10.1186/s40814-025-01706-5

**Published:** 2025-10-28

**Authors:** Candice Richardson, Cindy Chan, Emily Macgregor, Crystal Hare, Breffni Hannon, SarahRose Black, Evan Schneider, Daniel Buchman, Stella Wang, Valeria Rac, Lusine Abrahamyan, Ella Huszti, Rinat Nissim, Madeline Li, Camilla Zimmermann, Emma Hapke, Daniel Rosenbaum, Sarah Hales

**Affiliations:** 1 Centre for Mental Health, University Health Network, Toronto, Canada; 2 Psychedelic Psychotherapy Research Group, University Health Network, Toronto, Canada; 3 Department of Supportive Care, Princess Margaret Cancer Centre, Toronto, Canada; 4 Department of Medicine, Division of Palliative Medicine, University of Toronto, Toronto, Canada; 5 Department of Family & Community Medicine, Division of Palliative Care, University of Toronto, Toronto, Canada; 6 Temmy Latner Centre for Palliative Care, Mount Sinai Hospital, Toronto, Canada; 7 Centre for Addiction and Mental Health, Toronto, Canada; 8 Dalla Lana School of Public Health, University of Toronto, Toronto, Canada; 9 Joint Centre for Bioethics, University of Toronto, Toronto, Canada; 10 Krembil Research Institute, University Health Network, Toronto, Canada; 11 Biostatistics Research Unit, University Health Network, Toronto, Canada; 12 Toronto Health Economics and Technology Assessment (THETA) Collaborative, Toronto General Hospital, University Health Network, Toronto, Canada; 13 Department of Psychiatry, University of Toronto, Toronto, Canada

**Keywords:** Psilocybin, Psychotherapy, End-of-life, Depression, Palliative care, Attachment theory, Existential theory, Relational theory, Open-label, Pilot, Feasibility

## Abstract

**Background:**

Individuals with advanced cancer often experience high levels of distress for which there are few standardized treatment approaches. Our multidisciplinary team has combined existing evidence-based approaches into Psilocybin-assisted Existential, Attachment, and RelationaL (PEARL) therapy. PEARL therapy combines elements from psilocybin-assisted psychotherapy, including preparatory therapy sessions, a high-dose psilocybin session, and integration sessions, with important elements from evidence-based psychotherapies designed for patients with advanced cancer.

**Method:**

This open-label, single-arm clinical trial will assess the acceptability, feasibility, and safety of PEARL therapy among 15 patients with advanced cancer, using qualitative and quantitative methodologies. Participants will complete self-report questionnaires at four time points pre- and post-intervention, as well as a qualitative interview one month after PEARL completion. Feasibility will be evaluated in terms of recruitment, retention, and adherence rates, while safety will be assessed based on the number of participants experiencing no serious adverse events.

**Discussion:**

This study will yield important information about the acceptability and feasibility of PEARL therapy and contribute to growing research around the efficacy of psychedelic-assisted therapies. PEARL therapy has the potential to improve quality of life among those with advanced disease, and careful research is needed to guide public policy, legislation, therapist training, and clinical guidelines.

**Trial registration:**

NCT06416085; 2024–07-16.

**Supplementary Information:**

The online version contains supplementary material available at 10.1186/s40814-025-01706-5.

## Background

Advanced and life-threatening cancer brings multiple challenges, including progressive disability and physical burden of disease, complex treatment decisions, changes in social roles and relationships, and re-evaluation of sources of personal meaning and values. Additionally, the threat of mortality may evoke anticipatory fears related to the possibility of future dependency, isolation, physical suffering, dying, and death. The distress associated with advanced disease may be experienced as psychiatric symptoms of depression, anxiety, posttraumatic stress [1, 2], or as other forms of distress, such as demoralization, hopelessness, death anxiety, existential distress, spiritual suffering, the desire for hastened death, or loss of a sense of dignity [3]. The recent shift to earlier introduction of palliative care for those with advanced disease has been shown to improve quality of life and survival [4, 5], and yet, compared to management of physical symptoms, there remain relatively few standardized treatment approaches for psychological, social, and existential suffering [6].

A breadth of unique issues should be considered in the psychotherapeutic treatment of those with advanced or life-threatening disease. These include varying physical status, fluctuations in ability to engage in psychotherapy, practical and resource needs, relational changes imposed by the illness, needs of family and wider support systems, and differences in mortality salience and readiness to explore death-related concerns. A psychotherapeutic framework informed by existential, attachment, and relational theories may address this range of challenges while allowing for an individualized approach. An example of a psychotherapy informed by these theories is CALM (Managing Cancer And Living Meaningfully) therapy [7, 8], developed and researched previously by members of our team. CALM is a brief, semi-structured, manualized, individual psychotherapy for patients with advanced cancer and their loved ones. Within CALM, the general therapeutic strategy is to facilitate mentalization, which is the capacity to reflect on feeling states, to distinguish them from literal facts, and to accept the possibility of multiple perspectives on events in order to support “double awareness,” which is the ability to prepare for end of life while continuing to live in and enjoy the present [9, 10]. This approach has been shown to reduce psychological distress and improve end-of-life preparation in the advanced cancer population [11] and is a recommended standard for early psychological palliative care [12].

Preliminary research has demonstrated the potential of psilocybin-assisted psychotherapy in treating psychological distress experienced by patients with cancer [13, 14]. Psilocybin is a psychedelic compound that occurs naturally in the *Psilocybe* genus of mushrooms and exerts its primary effects via agonism at the serotonin 5-hydroxytryptamine type 2A (5-HT2A) receptor. At adequate doses, psilocybin induces profound alterations in thought, perception, and emotion, together with experiences of ego dissolution or mystical-type experiences [15]. Contemporary therapeutic use of psilocybin is usually paired with psychotherapy or a psychological support paradigm, whereby participants undergo one or a small number of guided, high-dose psilocybin treatment sessions preceded by preparatory sessions and followed by integration sessions [16]. Recent research has demonstrated, with large effect sizes, the benefits of psilocybin-assisted psychotherapy for reducing end-of-life distress in patients with advanced disease. Ross et al. [17] conducted a systematic review of clinical trials using psychedelic treatment in patients with serious or terminal illnesses and associated psychiatric issues. Ten studies with a total of 445 participants met the inclusion criteria. Combined data from the six open-label trials included suggested that psychedelic-assisted psychotherapy is associated with reduced cancer-related depression, anxiety, and fear of death. Furthermore, the four randomized controlled trials (RCTs), which predominantly used psilocybin, showed that psychedelic-assisted psychotherapy produced rapid and sustained improvements in cancer-related psychological and existential distress with large effect sizes [18–21]. Reiche et al. [22] conducted a similar systematic review, concluding that patients with life-threatening diseases, and associated depression and anxiety, can benefit from the antidepressant and anxiolytic properties of serotonergic hallucinogens, with some studies also reporting reduced fear of death and enhanced quality of life. These effects appear to be rapid and may be sustained for months to years following a single high-dose drug session [23–26].

A more recent systematic review [24] included an additional contemporary RCT exploring MDMA (3,4-methylenedioxymethamphetamine)-assisted psychotherapy for the treatment of anxiety and other psychological distress associated with life-threatening illness [27], along with a non-controlled trial of psilocybin-assisted therapy, delivered in a group setting, for the treatment of demoralization among long-term AIDS survivor men [28]; this review reached the same conclusions as Ross et al. [17] and Reiche et al. [22], while noting that most of the included studies lacked detailed information about the psychotherapeutic approaches employed, as well as other nonpharmacologic or contextual factors such as music playlists used [24]. Finally, two recent trials have demonstrated the feasibility, safety, and possible efficacy of psilocybin-assisted group therapy for patients with cancer [25, 26]. Beaussant et al. [29] conducted qualitative exit interviews among 28 participants in the Agarwal et al. [26] clinical trial and found that participants held positive attitudes toward the acceptability of group-based psilocybin-assisted therapy.

Combining psilocybin-assisted psychotherapy protocols [18, 20], with an existential, attachment, and relationally informed psychotherapeutic approach, we have developed a novel psilocybin-assisted psychotherapy intervention for patients with advanced cancer. This new intervention is called Psilocybin-assisted Existential, Attachment, and RelationaL (PEARL) therapy. The rationale for this feasibility trial is three-fold. Firstly, while it is well understood that the psychological effects of psilocybin are highly context-dependent [30], the psychotherapeutic components of existing psilocybin-assisted psychotherapy interventions have received little attention [24] and have not been informed by contemporary research regarding effective psychotherapeutic care of this population. Thus, defining potential therapeutic protocols, including the amount and type of psychotherapy, has been identified as a research priority [31, 32]. Second, patient preferences and expectancies may influence outcomes with psychedelics, and in most studies to date, patients have been motivated to engage, have self-referred, and in some cases have qualified for participation, despite having cancer in remission [18, 20]. It is unclear how feasible or acceptable PEARL might be for patients living with advanced cancer recruited within a comprehensive cancer center. Finally, the most relevant clinical outcomes remain unclear, with studies to date focusing on a variety of distress outcomes [31]. This protocol describes a feasibility and acceptability trial, the results of which will inform further intervention refinement and a randomized controlled trial (RCT) examining the efficacy of PEARL therapy.

### Objectives

- 1.a. To assess the feasibility of PEARL therapy with attention to recruitment, retention, and adherence.
- 1.b. To assess the acceptability of PEARL therapy from the perspective of patients.
- 1.c. To assess the safety of PEARL therapy.
- 2.a. To explore the preliminary efficacy of PEARL regarding outcomes including death-related distress, depressive symptoms, anxiety, demoralization, spiritual wellbeing, quality of life, and desire for hastened death.
- 2.b. To explore patient perspectives on the clinical relevance of these measurable outcomes.

## Methods

### Study design and setting

This is a single-center, open-label, one-arm, clinical trial using both qualitative and quantitative methodologies. Participants will be recruited from Princess Margaret Cancer Centre (PM), Canada’s largest comprehensive cancer treatment and research center, which includes a large Department of Supportive Care that provides outpatient psychosocial oncology and palliative care services.

### Participants

Participant inclusion criteria are as follows: (i) > 18 years of age, (ii) ability to speak and read English, (iii) no cognitive impairment, (iv) confirmed diagnosis of stage IV solid tumor/sarcoma/melanoma/lymphoma/endocrine cancer with expected survival of greater than 6 months, (v) at least mild depressive symptoms (PHQ-9 > 8), (vi) normal hepatic and renal function (INR < 1.5, AST/ALT < 2 × ULN, normal range bilirubin, PLT ≥ 150, eGFR > 45), and (vii) interest and ability to participate in the PEARL intervention as outlined. Furthermore, if sexually active and able to become pregnant, participants must be using effective birth control and cannot be pregnant or nursing for the duration of the study. Regarding medications and substances, for 1 week prior to the psilocybin session, participants must agree to refrain from (i) using any prescription medications not approved by the team and (ii) using any non-prescription medication, nutritional, or herbal supplement not approved by the team (exceptions include acetaminophen, non-steroidal anti-inflammatories, and common doses of vitamins and minerals). Additionally, participants must refrain from (i) taking any as needed medication on the morning of the psilocybin session (with the exception of daily and as needed opioid medication); (ii) using nicotine for at least 2 h prior to, and 7 h after, psilocybin administration; and (iii) using any psychoactive drugs, including alcohol, within 24 h of psilocybin administration.

Participant exclusion criteria include (i) cancer of, or metastasis to, the brain; (ii) symptoms consistent with delirium, psychosis, or other symptoms judged to be incompatible with establishment of rapport or safe exposure to psilocybin; (iii) history of past intolerability to psychedelics; (iv) past/present psychiatric diagnoses of bipolar disorder, psychotic disorder, active substance use disorder, or suicidality (as distinguished from desire for hastened death or readiness for death, per the discretion of the study team); (v) first degree relative with primary psychotic disorder if participant is under age 30; (vi) severe hypertension (SBP > 140 or DBP > 90) based on two readings on the same day; (vii) known paraneoplastic syndrome or “ectopic” hormone production by the primary tumor if incompatible with psilocybin, determined in consultation with the study palliative care physician; (viii) cardiovascular conditions including uncontrolled hypertension, angina, clinically significant ECG abnormality, transient ischemic attack in the last six months, stroke, peripheral or pulmonary vascular disease; (ix) uncontrolled epilepsy or history of seizures in the past 6 months; (x) gastrointestinal bleed in the past 6 months; (xi) participants with diabetes who are unable to skip a meal, require medication administration more than twice daily, or who have had symptomatic hypoglycemia within the prior 30 days; (xii) use of other investigational agents, psychoactive prescription medications (e.g., benzodiazepines, lithium, SSRIs), medications with primary pharmacological effect on 5-HT2A receptors (e.g., olanzapine), monoamine oxidase inhibitors, potent metabolic inducers (e.g., rifamycin, rifampin, rifabutin, rifapentine, carbamazepine, phenytoin, phenobarbital, nevirapine, efavirenz, paclitaxel, dexamethasone, St John’s wort), or inhibitors (e.g., HIV protease inhibitors, itraconazole, ketoconazole, erythromycin, clarithromycin, troleandomycin). In suitable patients, if safe to do so, contraindicated medications may be tapered prior to the start of the intervention.

### PEARL intervention

All participants will receive the PEARL therapy intervention, which consists of eight psychotherapy sessions delivered over a 7-week period. Each participant will be assigned a primary therapist for all sessions, and a secondary therapist will be present at the first psychotherapy session as well as the final preparatory, psilocybin, and first integration sessions. The PEARL foundations include existential, attachment, and relational theories, and the process is semi-structured, guided by the patient’s concerns, with a focus on joint creation of meaning between therapist(s) and patient, and facilitation of mentalization. PEARL therapists are PEARL-trained psychiatrists, psychologists, and spiritual care providers with expertise in treating patients with advanced cancer. Each therapy dyad will include at least one psychiatrist.

#### Initial psychotherapy sessions

Three initial sessions, 75 min in length, are aimed to develop a therapeutic alliance and explore the determinants of the patient’s distress considering four broad domains: (1) the physical experience (disease, symptoms, and management), (2) the relational context (changes in self-concept and relationships with close others), (3) spirituality and sources of meaning and purpose, and (4) their orientation to the future, hope, and mortality. A family caregiver will be invited to participate in one or more of these sessions to explore and support family functioning in the context of cancer and to prepare them for the psilocybin session, answer questions, and help them understand how to support the participant after the session.

#### Preparatory session

This 90-min session will be conducted 1 week before the psilocybin session within the treatment setting (a relaxed, calming space with controllable lighting and temperature, with availability of pre-selected recorded music via headphones and a surround sound speaker system) and will focus on preparation for the psilocybin session.

#### Psilocybin session

This session will last an average of 6–8 h. The patient will ingest a 25-mg psilocybin capsule administered by the study physician(s) within the treatment setting. Both therapists will act as supports or guides according to principles of psychedelic-assisted psychotherapy (the use of eye shades, music, inner-directed with therapists providing support as needed and facilitating a sense of safety) [33].

#### Integration session

This 90-min session occurring the day after the psilocybin session will support reflection on the experiences of and insights from the psilocybin session, understanding these in the context of the patient’s individual psychology, and considering their impact on the four content domains: (1) the physical experience (disease, symptoms, and management); (2) the relational context (changes in self-concept and relationships with close others); (3) spirituality and sources of meaning and purpose; and (4) their orientation to the future, hope, and mortality). Some attention will be paid to helping the participant continue their integration process on their own. A family caregiver will be invited to attend a portion of this session.

#### Follow-up sessions

Two further psychotherapy sessions, similar in nature and duration to the initial integration session, with the primary therapist will take place weekly to continue to explore and integrate the experience.

#### Investigational agent

Participants will ingest one 25-mg capsule of synthetic psilocybin at the beginning of the dosing session. Oral psilocybin, 25 mg capsules, are within the dosing range previously shown to be safe and well-tolerated in other clinical trials in this population [17, 18, 26, 29]. Psilocybin capsules are manufactured by Usona Institute under a collaborative agreement from their Investigational Drug and Material Supply Program.

#### Discontinuation criteria

Participants can withdraw from treatment or withdraw from the study at any time for any reason. The investigator may withdraw a participant if (a) the participant develops any conditions listed within the exclusion criteria (e.g., medical or psychiatric diagnosis/symptoms) that affect the safety of the participant, (b) the participant cannot comply with elements of the protocol that are critical for safety, or (c) in their clinical judgment, it is in the best interest of the participant. If the clinical team decides to terminate a participant from the study, they will explain to the participant, document the reason for removal, and ensure the participant has an appropriate follow-up support plan.

### Study procedures

**Fig. 1:**
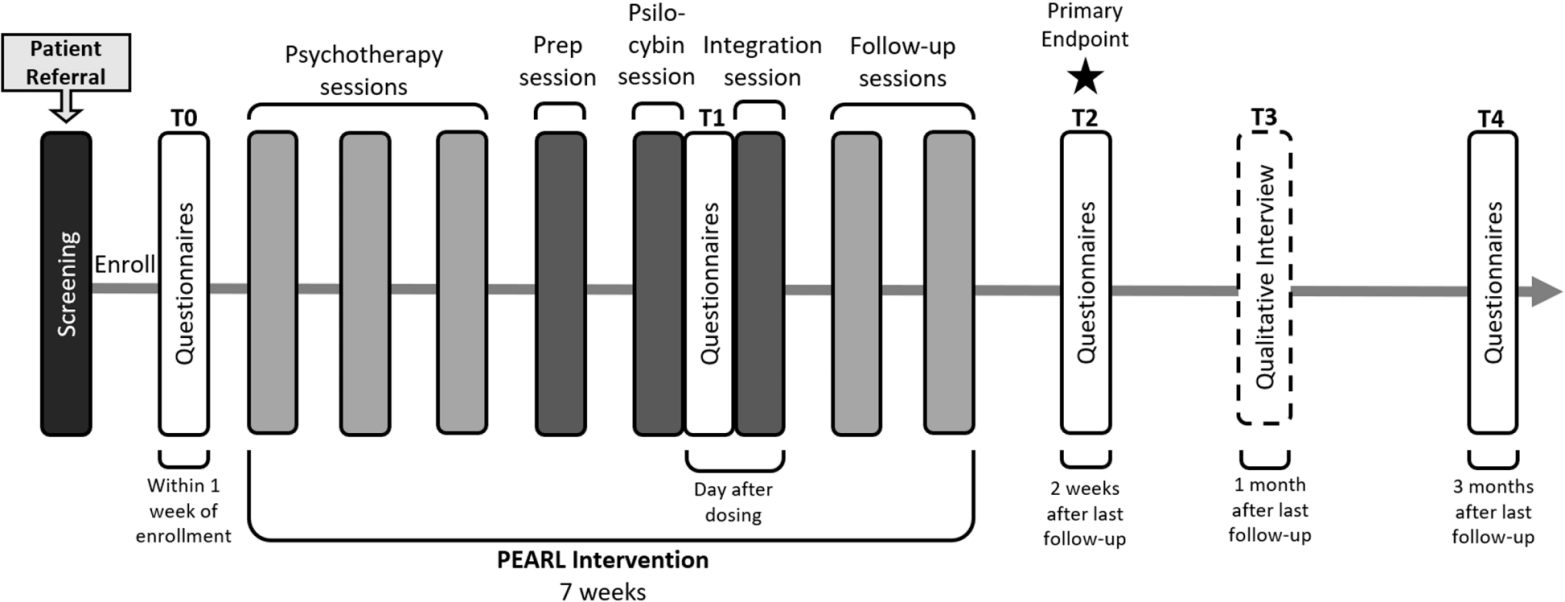
Study schedule

**Fig. 2:**
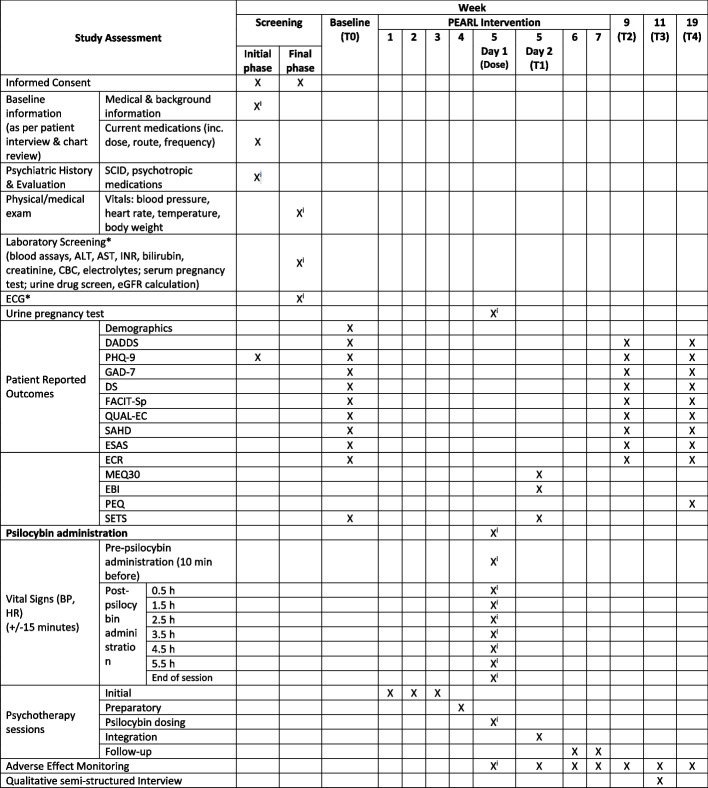
Study calendar

The study schedule is organized as follows (see Fig. 1). Potentially eligible participants will be referred from outpatient clinics at PM. Research staff will contact potential participants to obtain consent for initial screening, which involves medical chart review, laboratory investigations, and psychiatric assessment. Participants assessed as potentially eligible will continue to the final screening procedures once informed consent for the main study is obtained. If participants meet all inclusion/exclusion criteria, they will be enrolled in the trial and complete baseline questionnaires (T0) within 1 week. Participants will begin the PEARL intervention within 1 to 2 weeks of T0. On the day following the psilocybin session, participants will complete questionnaires to assess the quality of the experience (T1). Participants will complete follow-up questionnaires 2 weeks (T2) following PEARL completion, a qualitative interview 1 month following PEARL completion (T3), and follow-up questionnaires 3 months following PEARL completion (T4). The assessments, intervention components, and questionnaires completed at each time point are outlined in the study calendar (Fig. 2).

### Sample size

We have chosen a target sample size of 15 participants. There is little consensus in the literature regarding sample sizes required for single-arm feasibility and acceptability studies, and guidance varies depending on study design and goals, as well as pragmatic issues such as the value of information, study cost, and other constraints [34]. In terms of human resources, time, and the price of psilocybin (a controlled substance that is expensive to purchase and import into Canada), this is a costly complex intervention to deliver, and therefore, the smallest meaningful sample size was preferred. The likely sample size of any future definitive RCT also informed our decision. While this is not a pilot RCT, it has been suggested that the size of a pilot trial should be informed by the size of the future definitive RCT with possible sample sizes of 10 to 75 depending on the potential effect size (from large to very small) [35]. Given the large effect sizes reported following previous psilocybin-assisted psychotherapy RCTs in this population (treatment arms in these trials ranged from 16 [20] to 29 [18]), a sample size of 15 for this single-arm study was deemed reasonable. Finally, our primary objectives include examination of both quantitative and qualitative indicators of feasibility and acceptability. Considering quantitative indicators, with a sample size of 15, we will be able to estimate, with 95% confidence, the anticipated recruitment rate of 40% with 25% absolute precision and the anticipated retention rate of 75% with 20% absolute precision. Considering qualitative indicators, samples of less than 20 have been deemed suitable for qualitative feasibility research [36].

### Treatment integrity

The treatment integrity of the PEARL intervention will be ensured by means of (1) reference to the PEARL Treatment Manual, which defines the characteristics and sequencing of the intervention and provides examples of approaches to respond to disruptions or deviations and (2) regular supervision of PEARL therapists by PEARL developers, which will include review of selected video-recorded sessions. Evaluation will be conducted by PEARL developers, using the PEARL Treatment Integrity Scale (see Additional file 1) adapted from CALM treatment integrity tools [8].

### Outcomes

#### Feasibility

Feasibility will be evaluated in terms of recruitment, retention, and adherence. Recruitment will be assessed by capturing the number of patients who consent to participate after referral and screening. Retention will be assessed by tracking completion of study measures across all time points. Adherence will be assessed by tracking the number of PEARL sessions completed.

#### Acceptability

Participants will be interviewed regarding their experiences with PEARL, including acceptability, and perceived positive and negative effects of the intervention, with a semi-structured interview guide (see Additional file 2). Interviews will be video recorded and professionally transcribed.

#### Safety

This study will utilize the Common Terminology Criteria for Adverse Events (CTCAE) version 5.0 [37] for toxicity and adverse event (AE) reporting. AEs attributed to psilocybin will be monitored for and recorded after the psilocybin session. During the psilocybin session, the Monitor Rating Questionnaire, adapted from the measure used by Griffiths et al. [18], will be completed by therapists to capture AEs including elevated heart rate and blood pressure, confusion, headache, nausea/vomiting, anxiety, and paranoia. Cardiovascular variables (heart rate and blood pressure) will be measured during the dosing session at the following time points: baseline, 30, 90, 150, 210, 270, and 330 min post-dose administration and at the end of the session with a ± 15 min window. Vital signs will be measured more frequently if there are abnormal readings of heart rate and blood pressure, or if there is chest pain, shortness of breath, neurological symptoms, or any other signs or symptoms that may be indicative of a medical complication.

Serious adverse events (SAEs) will be tracked until study completion and will be defined as any adverse drug experience that results in death, is life-threatening, requires hospital admission, results in persistent or significant disability, or may jeopardize the participant or necessitate medical intervention to prevent one of the aforementioned events.

#### Additional data collection

The following demographic information will be collected via self-report: age, gender, sexual orientation, ethnicity, level of education, marital status, household income, and living arrangements. Medical and background information including cancer type and stage, length of illness, previous psychiatric and medical diagnoses, current medications, and past use of psychedelic medicines will be gathered via medical interview, physical examination, laboratory screening, and chart review. The Structured Clinical Interview for DSM Disorders (SCID) depression module will be administered to accurately assess for the current diagnosis of depression during screening.

### Data analysis

#### Feasibility (Objective 1.a)

Based on guidance for conducting feasibility trials [38, 39], we have established clear progression criteria (green/proceed, amber/amend, red/stop), informed by previous research on similar interventions in similar populations, to determine if the study findings indicate that it would be feasible to examine the efficacy of PEARL therapy in a larger RCT. All progression criteria will be summarized using descriptive statistics.

*Recruitment* criteria are based on the percentage of interested and eligible patients who consent to participate. Green (progression to RCT) will be supported at or above 40%, amber (minor protocol amendment before progression) at 10–40%, and red (significant protocol amendment before progression) at or below 10%. We are aiming for consent rates as high as Griffiths et al. [18] documented in their psilocybin-assisted psychotherapy trial among cancer patients (41%). Given that the PEARL protocol is more demanding and time-consuming for patients, we will allow for a slightly lower consent rate.

*Retention* criteria are based on the percentage of participants completing primary endpoint (T3) measures. Green will be supported at or above 75%, amber at 40–75%, and red at or below 40%. Criteria are based on 18–26% loss to follow-up rates in similar studies [11, 17, 18], and we will allow for loss on the higher end given the time commitment and demands of the PEARL protocol.

*Adherence* criteria are based on the percentage of participants completing all eight PEARL intervention sessions. Green will be supported at or above 70%, amber at 40–70%, and red at or below 40%. Criteria are based on the CALM RCT at PM, which achieved 77.5% adherence (completion of at least 3 sessions) among a similar target population [11]. We have chosen a lower threshold given that we are planning more sessions within a shorter time frame.

#### Acceptability (Objective 1.b)

Transcripts from qualitative interviews will be systematically coded using thematic analysis [40]. An initial set of themes regarding acceptability will be coded to form a tentative coding scheme. This scheme will be applied to new transcripts and revised to adjust for new information. Analysis will be conducted in an iterative process. An audit trail consisting of a detailed chronology of data collection and analytical decisions will be maintained, and regular team meetings will be conducted to discuss all phases of the analysis to resolve discrepancies and reach consensus.

#### Safety (Objective 1.c)

Safety criteria are based on the number of participants experiencing no SAEs throughout the course of the trial. Green will be supported at 100%, amber at 85–100%, and red at or below 85%. Similar trials using psilocybin-assisted psychotherapy [18, 20] reported no SAEs. While we anticipate the same, we will allow for a slightly lower threshold given that our patient population may be more ill than the population recruited in previous studies.

#### Efficacy outcomes (Objective 2.a)

The following outcome measures will be collected pre- and post-intervention at the time points indicated in the study calendar: death-related distress (Death and Dying Distress Scale (DADDS) [1, 14, 41]), depressive symptoms (Patient Health Questionnaire-9 (PHQ-9) [42]), anxiety symptoms (Generalized Anxiety Disorder scale (GAD-7) [43]), physical symptoms (Edmonton Symptom Assessment Scale (ESAS) [44]), demoralization symptoms (Demoralization Scale (DS) [45]), attachment style (the Experiences in Close Relationships Inventory (ECR) [46]), spiritual wellbeing (Functional Assessment of Chronic Illness Therapy-Spiritual Well-Being Scale (FACIT-Sp) [47]), quality of life (Quality of Life at the End of Life-Cancer Scale (QUAL-EC) [48]), attitudes toward hastened death (Schedule of Attitudes Toward Hastened Death (SAHD) [49]), and treatment expectancy (Stanford Expectation of Treatment Scale (SETS) [50]). Outcome measure packages will take an average of 25 min to complete. In our previous intervention studies in this population, measure packages of similar length were found acceptable to participants.

The following measures will only be completed post-dosing session to capture phenomena related to the psilocybin experience: quality of acute drug experience (Mystical Experience Questionnaire (MEQ30) [51]), persisting effects (three questions from the Persisting Effect Questionnaire (PEQ) [52]: (1) how personally meaningful was the experience, (2) how spiritually significant was the experience, and (3) did the experience change your sense of well-being or life satisfaction and emotional breakthrough (Emotional Breakthrough Inventory (EBI) [53]).

As a feasibility study, we consider all efficacy analyses to be exploratory and therefore will not be interpreting or disseminating the study outcome results as definitive and will not rely on any imputation methods. Outcome measures will be summarized with descriptive statistics, and dependent *t*-tests will be used to examine change over time, comparing each endpoint assessment to baseline with an intention-to-treat (ITT) analysis.

#### Relevance of outcomes (Objective 2.b)

To explore patient perspectives on the clinical relevance of potential therapy outcomes, questions about the outcome measures have been included in the semi-structured qualitative interview guide (Additional file 2). Transcripts will be systematically coded and analyzed using thematic analysis as described in Objective 1.b.

#### Data management and trial oversight

All data obtained in the clinical trial will be reported on electronic case report forms (eCRFs). All data will be source verified prior to publication; the investigator will review the data and electronically sign the eCRFs to acknowledge agreement with the data entered.

Data collected from all enrolled participants will be included in the analysis. If a participant withdraws or is terminated from the study, data collected up to that point will be included in analyses unless the participant specifically requests their data not be included. Any planned or unplanned deviation(s) from the original statistical plan will be described in the final report. Study termination and follow-up will be performed in compliance with the conditions set forth in the International Conference on Harmonisation (ICH) sixth efficacy publication on Good Clinical Practice. As per Health Canada regulations, all original records will be maintained for 15 years after study completion.

A trial steering committee composed of the principal investigators and external experts in clinical trials will provide oversight of study progress and procedures, ensure adherence to protocols and standards, and advise on ethical issues. Data safety monitoring will be performed by the PM Drug Development Program Central Office. Trial data in eCRFs will be monitored on an ongoing basis for unexpected or adverse events, and quality assurance measures will be performed.

## Discussion

Psilocybin remains a controlled substance not approved for clinical use in Canada in any population, while interest in clinical uses of psilocybin and other psychedelics is growing both inside and outside health care. There is thus an urgent need for rigorous research to inform policy, training, and clinical guidelines. At the same time, there is growing awareness of the numerous clinically important components of psychedelic-assisted psychotherapy that require attention [32, 54]. These include the sourcing, importing, and administration of the psychedelic medicine, and then the careful monitoring of its effects. Additionally, there are issues related to the selection and standardization of the psychotherapy component, therapist selection, training and supervision, and evaluation of treatment integrity. Finally, providing this type of intervention, and conducting this research, in a population living with advanced cancer, which may be physically and psychologically vulnerable, could create significant challenges to recruitment, retention, adherence, and acceptability. Together, these issues emphasize the importance of this feasibility and acceptability study prior to attempting further research in a larger randomized controlled trial.

There is increasing recognition that qualitative and quantitative methods are useful at this stage of trial development [55], and this trial draws on both methodologies. Qualitative feasibility studies are growing in number, and there are clearer guidelines around conducting qualitative research of this type [36, 56]. This approach is particularly suited to interventions such as PEARL that include a non-pharmacological component, which is modifiable based on participant feedback.

Recent reviews of trials in psychedelic medicine have highlighted a variety of issues including the lack of published protocols and lack of attention to the psychotherapeutic component of the intervention and psychotherapy fidelity [32, 57]. The design of this feasibility and acceptability trial, and publishing the protocol ahead of trial completion, represents an attempt to respond to these suggestions and increase the trust in, and value of, the associated evidence.

## Supplementary Information

Additional file 1: PEARL Treatment Integrity Scale.

Additional file 2. Interview Guide: PEARL Semi-structured Interview Guide V1.0.

Additional file 3. PEARL Study Consent Form V3.0.

Additional file 4. SPIRIT 2013 Checklist: Recommended items to address in a clinical trial protocol and related documents.

## Data Availability

Data sharing is not applicable to this article as no datasets were generated or analyzed during the current study.

## Abbreviations

PEARL: Psilocybin-assisted Existential, Attachment, and RelationaL
CALM: Managing Cancer And Living Meaningfully
5-HT2A: 5-Hydroxytryptamine type 2A
RCT: Randomized controlled trial
MDMA: 3,4-Methylenedioxymethamphetamine
AIDS: Acquired immunodeficiency syndrome
PM: Princess Margaret Cancer Center
INR: International normalized ratio
AST: Aspartate aminotransferase
ALT: Alanine transaminase
ULN: Upper limit of normal
PLT: Platelets
eGFR: Estimated glomerular filtration rate
SBP: Systolic blood pressure
DBP: Diastolic blood pressure
SSRI: Selective serotonin reuptake inhibitor
HIV: Human immunodeficiency virus
CTCAE: Common Terminology Criteria for Adverse Events version 5.0
AE: Adverse event
SAE: Serious adverse event
SCID: Structured Clinical Interview for DSM Disorders
DSM: Diagnostic and Statistical Manual of Mental Disorders
DADDS: Death and Dying Distress Scale
PHQ-9: Patient Health Questionnaire-9
GAD-7: Generalized Anxiety Disorder scale
ESAS: Edmonton Symptom Assessment Scale
DS: Demoralization Scale
ECR: Experiences in Close Relationships Inventory
FACIT-Sp: Functional Assessment of Chronic Illness Therapy-Spiritual Well-Being Scale
QUAL-EC: Quality of Life at the End of Life-Cancer Scale
SAHD: Schedule of Attitudes Towards Hastened Death
SETS: Stanford Expectation of Treatment Scale
MEQ30: Mystical Experience Questionnaire
PEQ: Persisting Effect Questionnaire
EBI: Emotional Breakthrough Inventory
ITT: Intention to treat
eCRFs: Electronic case report forms
ICH: International Conference on Harmonisation

## References

[1] Lo C, Hales S, Zimmermann C, Gagliese L, Rydall A, Rodin G. Measuring death-related anxiety in advanced cancer: preliminary psychometrics of the death and dying distress scale. J Pediatr Hematol Oncol. 2011;33(Suppl 2):S140–5. 21952572 10.1097/MPH.0b013e318230e1fd

[2] Rodin G, Lo C, Mikulincer M, Donner A, Gagliese L, Zimmermann C. Pathways to distress: the multiple determinants of depression, hopelessness, and the desire for hastened death in metastatic cancer patients. Soc Sci Med. 2009;68(3):562–9. 19059687 10.1016/j.socscimed.2008.10.037

[3] Vehling S, Philipp R. Existential distress and meaning-focused interventions in cancer survivorship. Curr Opin Support Palliat Care. 2018;12(1):46–51. 29251694 10.1097/SPC.0000000000000324

[4] Zimmermann C, Swami N, Krzyzanowska M, Hannon B, Leighl N, Oza A, et al. Early palliative care for patients with advanced cancer: a cluster-randomised controlled trial. Lancet. 2014;383(9930):1721–30. 10.1016/S0140-6736(13)62416-2. 24559581 10.1016/S0140-6736(13)62416-2

[5] Kavalieratos D, Corbelli J, Zhang D, Dionne-Odom JN, Ernecoff NC, Hanmer J, et al. Association between palliative care and patient and caregiver outcomes: a systematic review and meta-analysis. JAMA. 2016;316(20):2104–14. 10.1001/jama.2016.16840. 27893131 10.1001/jama.2016.16840 PMC5226373

[6] Galushko M, Romotzky V, Voltz R. Challenges in end-of-life communication. Curr Opin Support Palliat Care. 2012;6(3). Available from: https://journals.lww.com/co-supportiveandpalliativecare/Fulltext/2012/09000/Challenges_in_end_of_life_communication.11.aspx. 10.1097/SPC.0b013e328356ab72 22871981

[7] Hales S, Lo C, Rodin G. Managing Cancer And Living Meaningfully (CALM) therapy. In: Holland JC, Breitbart WS, Jacobsen PB, Loscalzo MJ, McCorkle R, Butow PN, editors. Psycho-Oncology. Third. New York, NY: Oxford University Press; 2015. p. 487–91.

[8] Rodin G, Hales S. Managing cancer and living meaningfully: an evidence-based intervention for cancer patients and their caregivers. New York, NY: Oxford University Press; 2021.

[9] Colosimo K, Nissim R, Pos AE, Hales S, Zimmermann C, Rodin G. “Double awareness” in psychotherapy for patients living with advanced cancer. Vol. 28, Journal of Psychotherapy Integration. Colosimo, Ken: Department of Psychology, York University, Behavioural Science Building, 4700 Keele Street, Toronto, ON, Canada, M3J 1P3, kennethc@yorku.ca: Educational Publishing Foundation; 2018. p. 125–40.

[10] Rodin G, Zimmermann C. Psychoanalytic reflections on mortality: a reconsideration. J Am Acad Psychoanal Dyn Psychiatry. 2008;36(1):181–96. 18399753 10.1521/jaap.2008.36.1.181

[11] Rodin G, Lo C, Rydall A, Shnall J, Malfitano C, Chiu A, et al. Managing cancer and living meaningfully (CALM): a randomized controlled trial of a psychological intervention for patients with advanced cancer. J Clin Oncol Off J Am Soc Clin Oncol. 2018;36(23):2422–32. 10.1200/JCO.2017.77.1097 PMC6085180 29958037

[12] Kaasa S, Loge JH, Aapro M, Albreht T, Anderson R, Bruera E, et al. Integration of oncology and palliative care: a Lancet Oncology Commission. Lancet Oncol. 2018;19(11):e588-653. 30344075 10.1016/S1470-2045(18)30415-7

[13] Fulton JJ, Newins AR, Porter LS, Ramos K. Psychotherapy targeting depression and anxiety for use in palliative care: a meta-analysis. J Palliat Med. 2018;21(7):1024–37. 10.1089/jpm.2017.0576. 29676960 10.1089/jpm.2017.0576

[14] Grossman CH, Brooker J, Michael N, Kissane D. Death anxiety interventions in patients with advanced cancer: a systematic review. Palliat Med. 2018;32(1):172–84. 28786328 10.1177/0269216317722123

[15] Nichols DE. Psychedelics. Barker EL, editor. Pharmacol Rev. 2016 Apr 1;68(2):264 LP – 355. Available from: http://pharmrev.aspetjournals.org/content/68/2/264.abstract. 10.1124/pr.115.011478 PMC4813425 26841800

[16] Gründer G, Brand M, Mertens LJ, Jungaberle H, Kärtner L, Scharf DJ, et al. Treatment with psychedelics is psychotherapy: beyond reductionism. Lancet Psychiatry. 2024;11(3):231–6. 10.1016/S2215-0366(23)00363-2. 38101439 10.1016/S2215-0366(23)00363-2

[17] Ross S. Therapeutic use of classic psychedelics to treat cancer-related psychiatric distress. Int Rev Psychiatry. 2018;30(4):317–30. 30102082 10.1080/09540261.2018.1482261

[18] Griffiths RR, Johnson MW, Carducci MA, Umbricht A, Richards WA, Richards BD, et al. Psilocybin produces substantial and sustained decreases in depression and anxiety in patients with life-threatening cancer: a randomized double-blind trial. J Psychopharmacol. 2016;30(12):1181–97. Available from: https://pubmed.ncbi.nlm.nih.gov/27909165. 10.1177/0269881116675513 PMC5367557 27909165

[19] Grob CS, Danforth AL, Chopra GS, Hagerty M, McKay CR, Halberstadt AL, et al. Pilot study of psilocybin treatment for anxiety in patients with advanced-stage cancer. Arch Gen Psychiatry. 2011;68(1):71–8. 10.1001/archgenpsychiatry.2010.116. 20819978 10.1001/archgenpsychiatry.2010.116

[20] Ross S, Bossis A, Guss J, Agin-Liebes G, Malone T, Cohen B, et al. Rapid and sustained symptom reduction following psilocybin treatment for anxiety and depression in patients with life-threatening cancer: a randomized controlled trial. J Psychopharmacol. 2016;30(12):1165–80. 27909164 10.1177/0269881116675512 PMC5367551

[21] Gasser P, Holstein D, Michel Y, Doblin R, Yazar-Klosinski B, Passie T, et al. Safety and efficacy of lysergic acid diethylamide-assisted psychotherapy for anxiety associated with life-threatening diseases. J Nerv Ment Dis. 2014;202(7):513–20. 24594678 10.1097/NMD.0000000000000113 PMC4086777

[22] Reiche S, Hermle L, Gutwinski S, Jungaberle H, Gasser P, Majić T. Serotonergic hallucinogens in the treatment of anxiety and depression in patients suffering from a life-threatening disease: a systematic review. Prog Neuropsychopharmacol Biol Psychiatry. 2018;81:1–10. 28947181 10.1016/j.pnpbp.2017.09.012

[23] Agin-Liebes GI, Malone T, Yalch MM, Mennenga SE, Ponté KL, Guss J, et al. Long-term follow-up of psilocybin-assisted psychotherapy for psychiatric and existential distress in patients with life-threatening cancer. J Psychopharmacol. 2020;34(2):155–66. 31916890 10.1177/0269881119897615

[24] Maia LO, Beaussant Y, Garcia ACM. The therapeutic potential of psychedelic-assisted therapies for symptom control in patients diagnosed with serious illness: a systematic review. J Pain Symptom Manage. 2022;63(6):e725–38. 35157985 10.1016/j.jpainsymman.2022.01.024

[25] Lewis BR, Garland EL, Byrne K, Durns T, Hendrick J, Beck A, et al. HOPE: a pilot study of psilocybin enhanced group psychotherapy in patients with cancer. J Pain Symptom Manage. 2023;66(3):258–69. 10.1016/j.jpainsymman.2023.06.006. 37302533 10.1016/j.jpainsymman.2023.06.006

[26] Agrawal M, Richards W, Beaussant Y, Shnayder S, Ameli R, Roddy K, et al. Psilocybin-assisted group therapy in patients with cancer diagnosed with a major depressive disorder. Cancer. 2024;130(7):1137–46. 10.1002/cncr.35010. 38105655 10.1002/cncr.35010

[27] Wolfson PE, Andries J, Feduccia AA, Jerome L, Wang JB, Williams E, et al. MDMA-assisted psychotherapy for treatment of anxiety and other psychological distress related to life-threatening illnesses: a randomized pilot study. Sci Rep. 2020;10(1):20442. 33235285 10.1038/s41598-020-75706-1 PMC7686344

[28] Anderson BT, Danforth A, Daroff PR, Stauffer C, Ekman E, Agin-Liebes G, et al. Psilocybin-assisted group therapy for demoralized older long-term AIDS survivor men: an open-label safety and feasibility pilot study. EClinicalMedicine. 2020;27:100538. 33150319 10.1016/j.eclinm.2020.100538 PMC7599297

[29] Beaussant Y, Tarbi E, Nigam K, Miner S, Sager Z, Sanders JJ, et al. Acceptability of psilocybin-assisted group therapy in patients with cancer and major depressive disorder: qualitative analysis. Cancer. 2024;130(7):1147–57. 10.1002/cncr.35024. 38105653 10.1002/cncr.35024

[30] Carhart-Harris RL, Roseman L, Haijen E, Erritzoe D, Watts R, Branchi I, et al. Psychedelics and the essential importance of context. J Psychopharmacol. 2018;32(7):725–31. 29446697 10.1177/0269881118754710

[31] Beaussant Y, Sanders J, Sager Z, Tulsky JA, Braun IM, Blinderman CD, et al. Defining the roles and research priorities for psychedelic-assisted therapies in patients with serious illness: expert clinicians’ and investigators’ perspectives. J Palliat Med. 2020;23(10):1323–34. 32233936 10.1089/jpm.2019.0603

[32] Hovmand OR, Poulsen ED, Arnfred S, Storebø OJ. Risk of bias in randomized clinical trials on psychedelic medicine: a systematic review. J Psychopharmacol. 2023;37(7):649–59. 37403379 10.1177/02698811231180276 PMC10350724

[33] American Psychedelic Practitioners Association and BrainFutures. Professional practice guidelines for psychedelic-assisted therapy. 2023. Available from: https://www.appa-us.org/standards-and-guidelines/professionalpracticeguidelines.

[34] Moore CG, Carter RE, Nietert PJ, Stewart PW. Recommendations for planning pilot studies in clinical and translational research. Clin Transl Sci. 2011;4(5):332–7. 22029804 10.1111/j.1752-8062.2011.00347.x PMC3203750

[35] Whitehead AL, Julious SA, Cooper CL, Campbell MJ. Estimating the sample size for a pilot randomised trial to minimise the overall trial sample size for the external pilot and main trial for a continuous outcome variable. Stat Methods Med Res. 2016;25(3):1057–73. 26092476 10.1177/0962280215588241 PMC4876429

[36] O’Cathain A, Hoddinott P, Lewin S, Thomas KJ, Young B, Adamson J, et al. Maximising the impact of qualitative research in feasibility studies for randomised controlled trials: guidance for researchers. Pilot Feasibility Stud. 2015;1(1):32. 10.1186/s40814-015-0026-y. 27965810 10.1186/s40814-015-0026-y PMC5154038

[37] U.S. Department of Health and Human Services. Common Terminology Criteria for Adverse Events (CTCAE) v5.0. 2017. Available from: https://ctep.cancer.gov/protocolDevelopment/electronic_applications/ctc.htm#ctc_50. Cited 2024 Jul 1.

[38] Thabane L, Ma J, Chu R, Cheng J, Ismaila A, Rios LP, et al. A tutorial on pilot studies: the what, why and how. BMC Med Res Methodol. 2010;10(1):1. 10.1186/1471-2288-10-1. 20053272 10.1186/1471-2288-10-1 PMC2824145

[39] Avery KNL, Williamson PR, Gamble C, Connell Francischetto E, Metcalfe C, Davidson P, et al. Informing efficient randomised controlled trials: exploration of challenges in developing progression criteria for internal pilot studies. BMJ Open. 2017;7(2):e013537. Available from: https://bmjopen.bmj.com/content/7/2/e013537.abstract. 28213598 10.1136/bmjopen-2016-013537 PMC5318608

[40] Braun V, Clarke V. Using thematic analysis in psychology. Qual Res Psychol. 2006;3(2):77–101. Available from: https://www.tandfonline.com/doi/abs/10.1191/1478088706qp063oa.

[41] Krause S, Rydall A, Hales S, Rodin G, Lo C. Initial validation of the death and dying distress scale for the assessment of death anxiety in patients with advanced cancer. J Pain Symptom Manage. 2015;49(1):126–34. 24878066 10.1016/j.jpainsymman.2014.04.012

[42] Kroenke K, Spitzer RL, Williams JB. The PHQ-9: validity of a brief depression severity measure. J Gen Intern Med. 2001;16(9):606–13. 11556941 10.1046/j.1525-1497.2001.016009606.x PMC1495268

[43] Spitzer RL, Kroenke K, Williams JBW, Löwe B. A brief measure for assessing generalized anxiety disorder: the GAD-7. Arch Intern Med. 2006;166(10):1092–7. 10.1001/archinte.166.10.1092. 16717171 10.1001/archinte.166.10.1092

[44] Bruera E, Kuehn N, Miller MJ, Selmser P, Macmillan K. The Edmonton symptom assessment system (ESAS): a simple method for the assessment of palliative care patients. J Palliat Care. 1991;7(2):6–9. 1714502

[45] Kissane DW, Wein S, Love A, Lee XQ, Kee PL, Clarke DM. The demoralization scale: a report of its development and preliminary validation. J Palliat Care. 2004;20(4):269–76. 15690829

[46] Brennan KA, Clark CL, Shaver PR. Self-report measurement of adult attachment: an integrative overview. Attachment theory and close relationships. New York, NY, US: The Guilford Press; 1998. p. 46–76.

[47] Peterman AH, Fitchett G, Brady MJ, Hernandez L, Cella D. Measuring spiritual well-being in people with cancer: the functional assessment of chronic illness therapy–Spiritual Well-being Scale (FACIT-Sp). Ann Behav Med. 2002;24(1):49–58. 12008794 10.1207/S15324796ABM2401_06

[48] Steinhauser KE, Clipp EC, Bosworth HB, McNeilly M, Christakis NA, Voils CI, et al. Measuring quality of life at the end of life: validation of the QUAL-E. Palliat Support Care. 2004;2(1):3–14. 16594230 10.1017/s1478951504040027

[49] Rosenfeld B, Breitbart W, Galietta M, Kaim M, Funesti-Esch J, Pessin H, et al. The schedule of attitudes toward hastened death: measuring desire for death in terminally ill cancer patients. Cancer. 2000;88(12):2868–75. 10870074 10.1002/1097-0142(20000615)88:12<2868::aid-cncr30>3.0.co;2-k

[50] Younger J, Gandhi V, Hubbard E, Mackey S. Development of the Stanford Expectations of Treatment Scale (SETS): a tool for measuring patient outcome expectancy in clinical trials. Clin Trials. 2012;9(6):767–76. 23169874 10.1177/1740774512465064

[51] Barrett FS, Johnson MW, Griffiths RR. Validation of the revised Mystical Experience Questionnaire in experimental sessions with psilocybin. J Psychopharmacol. 2015/10/06. 2015;29(11):1182–90. Available from: https://pubmed.ncbi.nlm.nih.gov/26442957. 10.1177/0269881115609019 PMC5203697 26442957

[52] Griffiths RR, Richards WA, McCann U, Jesse R. Psilocybin can occasion mystical-type experiences having substantial and sustained personal meaning and spiritual significance. Psychopharmacology. 2006;187(3):268–92. 16826400 10.1007/s00213-006-0457-5

[53] Roseman L, Haijen E, Idialu-Ikato K, Kaelen M, Watts R, Carhart-Harris R. Emotional breakthrough and psychedelics: validation of the emotional breakthrough inventory. J Psychopharmacol. 2019;33(9):1076–87. 31294673 10.1177/0269881119855974

[54] Rosenblat JD, Husain MI, Lee Y, McIntyre RS, Mansur RB, Castle D, et al. The Canadian Network for Mood and Anxiety Treatments (CANMAT) task force report: serotonergic psychedelic treatments for major depressive disorder. Can J Psychiatry. 2023;68(1):5–21. 35975555 10.1177/07067437221111371 PMC9720483

[55] Baldeh T, MacDonald T, Kosa SD, Lawson DO, Stalteri R, Olaiya OR, et al. More pilot trials could plan to use qualitative data: a meta-epidemiological study. Pilot Feasibility Stud. 2020;6(1):164. 10.1186/s40814-020-00712-z. 33292715 10.1186/s40814-020-00712-z PMC7597013

[56] Teresi JA, Yu X, Stewart AL, Hays RD. Guidelines for designing and evaluating feasibility pilot studies. Med Care. 2022;60(1):95–103. 34812790 10.1097/MLR.0000000000001664 PMC8849521

[57] Aday JS, Horton D, Fernandes-Osterhold G, O’Donovan A, Bradley ER, Rosen RC, et al. Psychedelic-assisted psychotherapy: where is the psychotherapy research? Psychopharmacology. 2024;241(8):1517–26. 38782821 10.1007/s00213-024-06620-x

