# Supplementary material for: Psilocybin-assisted Existential, Attachment and RelationaL (PEARL) therapy for patients with advanced cancer: protocol for a multi-method feasibility trial
Source: Pilot Feasibility Stud. 2025 Oct 28;11:126. doi: 10.1186/s40814-025-01706-5 (PMC12570686; doi:10.1186/s40814-025-01706-5)
Supplement: Supplementary file 1 — Additional file 1: PEARL Treatment Integrity Scale. [file 40814_2025_1706_MOESM1_ESM.pdf]

PEARL Treatment Integrity Scale

Therapist:____ (primary) & ____(secondary)
Case Number: ___
Case Supervision Date(s):______

This evaluation is completed on the basis of the case discussion in group supervision and the therapist's presentation of skills as demonstrated in those session(s). Each case presented will have one evaluation form completed. If a skill was not employed in situations which demanded it, then the skill should be rated negatively. If a skill was not employed because it was not applicable, the item can be left blank.

1: Needs improvement 2: Satisfactory 3: Excellent

The Therapeutic Relationship

___ Shows empathic understanding of patient experiences
___ Responds genuinely/honestly to patient thoughts and feelings
___ Acknowledges the realities of the patient's condition/situation
___ Maintains professional boundaries while engaging with patient experiences
___ Demonstrates investment/motivation/engagement in the therapeutic process

Modulating affect

___ Able to appropriately modulate the emotional state of the patient
___ Demonstrates comfort with emotional distress
___ Helps increase patient ability to think about/manage negative emotions/events
___ Validates the importance of both positive/ affirming and negative/challenging experiences as part of the therapeutic process

Shifting frame

___ Shift between supportive, exploratory and practical/problem solving therapeutic frames as necessary
___ Adjusts the content and timing of sessions based on the patient's physical and psychological state

Meaning-making

___ Promotes mentalizing (ability to consider multiple psychological responses to an event)
___ Offers potential explanations for the patient's pattern of distress, thoughts or behaviours
___ Makes judicious use of interpretations in the spirit of dialogue and exchange between therapist and patient in service of joint creation of meaning
___ Brackets own beliefs and goals to allow patient to derive their own meaning from the therapeutic experience

Rate the therapist's skills when addressing each content below, as applicable.

1: Need improvement 2: Satisfactory 3: Excellent

Symptom Management and Communication with Health Care Providers

___ Encourages better understanding of disease
___ Encourages patient's active involvement in medical care
___ Promotes patient consideration of treatment options
___ Supports communication with health care providers

Changes in self and relations with close others

___ Explores patient feelings about his/her life history
___ Validates patient's sense of worth in light of his/her accomplishments
___ Acknowledges disappointments or regrets that the patient has experiences
___ Explores the relational changes imposed by disease
___ Explores fears and anxieties about dependency and loss of autonomy
___ Encourages appropriate communication and support-giving/taking from close others

Spirituality or Sense of Meaning and Purpose

___ Explores the patient's spiritual beliefs and/or sense of meaning and purpose in life
___ Support understanding of the personal meaning of their experience of suffering and dying
___ Evaluates priorities and goals in the face of advanced disease
___ Helps to create new meanings regarding the patient's life trajectory, goals and suffering

Thinking of the Future, Hope and Mortality

___ Explores patient attitudes towards the future (i.e. hopes and fears about living and dying)
___ Allows expression of sadness and anxiety about the progression of disease
___ Explores feelings about death and dying
___ Promotes discussion of advance care planning
___ Helps to sustain realistic hope and engagement in life while acknowledging mortality

Preparation for Psilocybin Session

___ Creates a setting of safety and support
___ Introduces the concept that trust in one's own potential for meaning-making is a key part of this therapy
___ Explores patient fears, hopes, attitudes regarding psilocybin session
___ Provides psychoeducation about psilocybin session procedures and role of therapists
___ Discusses potential challenging experiences that may arise and how to approach them
___ Promotes the notion of the inner-directed approach ("experiential therapy"), i.e. orienting participants to idea that during dosing sessions they will be encouraged to "experience first ,interpret later" (language may vary)
___ Orients the participant to role of therapists on session day, including their presence and availability for support at any time, while also discussing importance of "sitting with" or "staying present to" difficult experiences

___ Discusses in clear language the optional use of touch for support or reassurance during dosing sessions, maintaining careful attunement to patients' reactions and level of comfort
___ As part of consent discussion around optional use of therapeutic touch, communicates clearly that touch is never sexual
___ Re: optional use of touch, provides clear orientation to "STOP" word

Psilocybin Session Guidance
___ Creates and communicates a setting of safety and support
___ Maintains non-intrusive stance with minimal interaction
___ Provides gentle reassurance and support as needed
___ Uses clear, simple language
___ Encourages patient to stay present with their immediate experience, including distressing thoughts/images/emotions, rather than avoiding them during session
___ Conveys a non-judgmental attitude towards the participants process and experience
___ Validates positive, affirming experiences or insights as important parts of the healing process
___ Ensures physical safety at all times (e.g., checking vital signs per the study protocol, assisting the participant to and from the toilet, using touch for purpose of containment if necessary and in accordance with consent discussions during preparation phase, etc.)
___ Makes judicious use of therapeutic touch, consistent with agreements and consent discussion that took place in preparatory sessions

Integration of Psilocybin Session
___ Facilitates participant-driven exploration of the psilocybin session's emotional, cognitive, and somatic experiential elements
___ As above, makes careful and judicious use of interpretations in the service of joint creation of meaning
___ Encourages reflection and synthesis of PEARL therapy session material
___ Invites exploration of both positive/affirming as well as negative/difficult elements of the psilocybin session, as appropriate
___ Provides education re: nature of ongoing integration and offers reassurance re: possibility of emergent emotional material which may evolve over time
___ On the day after the psilocybin session, encourages participant to create and take plenty of space for themselves to support the evolving emotional/healing process (i.e. not to take on too many responsibilities or strenuous activities, etc.)
___ Reviews with participant examples of common activities which may be incorporated into integration practices, e.g. journaling, meditation, yoga, time in nature, creative pursuits, or other activities the participant has identified as "resourcing" or important for their ongoing benefits and integration

Closure
___ Treatment ends with understanding of open-door policy; that therapist has not "given up" on patient

Supervisor Signature: ______________________ **Date**: ________________
