# Supplementary material for: Psilocybin-assisted Existential, Attachment and RelationaL (PEARL) therapy for patients with advanced cancer: protocol for a multi-method feasibility trial
Source: Pilot Feasibility Stud. 2025 Oct 28;11:126. doi: 10.1186/s40814-025-01706-5 (PMC12570686; doi:10.1186/s40814-025-01706-5)
Supplement: Supplementary file 2 — Additional file 2. Interview Guide: PEARL Semi-structured Interview Guide V1.0. [file 40814_2025_1706_MOESM2_ESM.pdf]

Interview Guide

PEARL Semi-structured Interview Guide V1.0

Draft date: 8 Feb 2022

Introduction

This interview is being conducted as part of a study to evaluate the PEARL intervention. My goal is to understand as deeply as I can your own experience as a participant in this intervention. Please don't feel rushed when answering my questions. We can always complete part of the interview today and schedule a second meeting to finish the interview at a later date, or we can reschedule the interview if you feel unable to fully participate today. Please know that you may stop the interview or discontinue your participation in the study at any time.

1. **How did you come to participate in the study?**

Probes:
- What problem/issue led you to be referred for or to seek out the PEARL intervention?
- What were your expectations, hopes or goals for participating in PEARL?
- Did you have any hesitations, concerns, or fears participating and if so, what were they?
- Were your healthcare team, family and/or friends supportive of your participation in PEARL?

2. **Can you tell me about your experience with PEARL?**

Probes:
- Was the experience what you expected? In what way?
- What were the most helpful/beneficial aspects?
- What were unhelpful/challenging aspects?
- What aspects were surprising to you?
- Would you recommend it to others? Why or why not? How would you describe it to others?

3. **What was your experience in the *preparatory phase* of the PEARL intervention? The preparatory phase includes the screening process, the assessment with the co-therapists and the first psychotherapy sessions prior to the psilocybin session.**

Probes:
- How were the PEARL sessions with the primary therapist?
- How was the preparatory session with both therapists?
- Did you feel adequately prepared for your dosing session? Why or why not?
- Do you have any suggestions for how we can help future participants be better prepared for this?

4. **What was your experience of the *psilocybin session*?**

Probes:

- What positive experiences did you have?
- What negative or challenging experiences did you have?
- What surprising experiences did you have?
- What was your experience of the musical component of the session?
- What was your experience in the hours and days following the psilocybin session?

5. **What was your experience of the integration sessions and study termination?**

Probes:

- Did you feel prepared for study termination?

6. **What was your experience of the therapists in the PEARL intervention?**

Probes:

- Experience with primary therapist present throughout the entire intervention
- Experience with secondary therapist present for assessment, preparation, dosing and integration

7. **How do you understand your primary caregivers and/or other family/support's experience of your participation in the PEARL study?**

Probes:

- Did your caregiver have certain expectations or hopes about your participation in PEARL
- Did your caregiver's have any hesitations or concerns about your participation in PEARL?
- Have you noticed any changes in relational patterns or dynamics since the PEARL intervention?
- Do you think your caregiver would be open to and would benefit from receiving their own session of psilocybin-assisted psychotherapy? Why or why not?

8. **How has your participation in the PEARL intervention impacted your life?**

Probes:

- Have there been any positive changes since participation?
- Have there been any negative changes since your participation?
- Based on your experience, how do you think psilocybin works to create such changes?

9. **Could you comment on any barriers/obstacles to your participation in this PEARL study (i.e. time commitment, screening process, etc.)?**

Probes:

- What about the timing in which the intervention was offered to you – e.g., do you think the intervention could have benefited you more if offered at a different time?

- Was the <u>length</u> of the program appropriate?
- Were you seeking or referred to <u>other forms of psychosocial support</u> while participating in this study? (if yes, ask for details)

10. In preparation for a larger trial, we are interested in your feedback on the self-report measures (i.e., questionnaires and rating scales) you completed. Which were most relevant to the PEARL experience? To your concerns prior to participation?

11. PEARL is designed to address four main areas of concern for many patients with advanced cancer. We are interested in how your experience in PEARL may have led to changes in these four areas. Did participation in PEARL lead to changes in any of these four domains? If so, how?

1. Your understanding of your illness, your symptoms, and relationship with health care providers
2. Your sense of identity and relationship with close others
3. Your spiritual wellbeing, or sense of meaning and purpose
4. Your attitude to the future, hope and mortality

12. Prior to participation in the PEARL, were you considering Medical Assistance in Dying (MAiD)?

<u>Probes</u>:
- If yes, can you tell us about your attitude toward MAiD prior to participating in PEARL?
- Was your decision to participate in PEARL related to helping make decisions about whether to undergo MAiD?
- Did participating in the PEARL protocol change your attitude toward MAID? How so?

13. Do you have any suggestions for how we can make this a better experience for participants?

14. (*Only for participants who withdrew from the study***) Can you tell us about your decision to withdraw from the study?**

<u>Probes</u>:
- What were your reasons for withdrawing?
- Why did you withdraw when you did?
- What were your expectations when you entered the study? What about your experience differed from expectations?
- Is there anything that would have made it possible to continue in the study?
