# Supplementary material for: Psilocybin-assisted Existential, Attachment and RelationaL (PEARL) therapy for patients with advanced cancer: protocol for a multi-method feasibility trial
Source: Pilot Feasibility Stud. 2025 Oct 28;11:126. doi: 10.1186/s40814-025-01706-5 (PMC12570686; doi:10.1186/s40814-025-01706-5)
Supplement: Supplementary file 3 — Additional file 3. PEARL Study Consent Form V3.0. [file 40814_2025_1706_MOESM3_ESM.pdf]

## PEARL STUDY CONSENT FORM

**Study Title:** Psilocybin-assisted Existential, Attachment and RelationaL (PEARL) Therapy for Patients with Advanced Cancer: A Phase II Open-Label Trial

**Principal Investigator:** Dr. Sarah Hales

**Contact Information:** 416-946-4501 x2551

**Funding Source:** Lotte & John Hecht Memorial Foundation

**Study Drug Provider:** Usona Institute

### Introduction:

You are being asked to take part in a research study. Please read the information about the study presented in this form. The form includes details on the study's risks and benefits that you should know before you decide if you would like to take part.

You should take as much time as you need to make your decision. You should ask the study doctor or study staff to explain anything that you do not understand and make sure that all of your questions have been answered before signing this consent form. Before you make your decision, feel free to talk about this study with anyone you wish including your friends, family, and family doctor.

Participation in this study is voluntary; you have the right to refuse to participate or withdraw from the study at any time.

### Background/Purpose:

Advanced cancer brings many challenges, including burdens on physical, social, and psychological health. There is currently little guidance for treating psychological symptoms associated with advanced disease such as depression, anxiety, post-traumatic stress, hopelessness, and spiritual suffering. Recent research has shown that psilocybin therapies have helped to reduce distress in patients with cancer.

Psilocybin-assisted Existential, Attachment and RelationaL (PEARL) therapy has been developed to help people manage the challenges of living with cancer, reduce death-related and other forms of distress, and promote well-being.

Psilocybin is a psychedelic substance (something that may change the way people see, hear, taste, smell or feel, and affect mood and thought), found naturally in certain species of mushrooms. In research studies, psilocybin is synthesized in a lab.

The use of psilocybin in patients with advanced cancer to reduce distress is ***experimental***. This means that Health Canada has not approved the sale or use of psilocybin in any population, but they have approved its use in this study.

***The purpose of this study*** is to find out whether a new medication-assisted psychotherapy, PEARL therapy, is practical, useful, and safe for patients with advanced cancer. The results of this study will help inform the type of therapy and support services that are helpful for patients with cancer. You are being asked to take part in this study because you have a diagnosis of advanced cancer and are experiencing anxiety, depression, and/or other forms of distress.

The usual treatment for patients with this diagnosis is psychotherapy designed for advanced illness, without psilocybin.
There will be about 15 people in this study, and it will take about 2 years to complete.

**Study Design:**

All participants will receive psilocybin-assisted therapy. This study involves a screening period, 8 **psychotherapy sessions** and 3 subsequent follow up interviews delivered over **3 months.**

During the **screening period,** the study team check whether a participant is eligible to receive the study drug.

**Psychotherapy** sessions involve:

- three (3) initial psychotherapy sessions
- one (1) preparatory session, which includes detailed education about what you can expect when you receive psilocybin
- one (1) day-long session, during which you will ingest a capsule containing psilocybin
- one (1) integration session, which involves reflection on the perspectives, insights, and experiences of the psilocybin session. A family caregiver will be invited to attend a portion of this session.
- two (2) follow-up psychotherapy sessions

**All study sessions and interview will be recorded** (both audio and video) so that the therapy team will have accurate records for data analysis. Treatment session recordings are helpful for researchers to analyze and ensure that all sessions are delivered according to the therapy and study guidelines. Video recordings also allow us to fully capture how participants feel during the sessions, behaviours and other nonverbal cues that participants may express during the psilocybin dosing session. Researchers will be able to look back into the dosing session and accurately draw a conclusion to answer study questions.

All treatment sessions will be recorded by the study therapists using either MS Teams Record and Transcribe or using the audio-visual (AV) recording equipment in the study therapy room.

Qualitative interviews will be recorded using the MS Teams Record and Transcribe function.

All sessions take place within a 7-week period.

**Study Visits and Procedures:**

The following procedures will be done during the study:

***Screening Period:***

After you have agreed to participate in the study, you will undergo evaluations to find out if you are eligible to receive the study drug. There will be in-person visits to the hospital, and virtual visits and phone calls from the study team. The screening period can take up to four (4-6) weeks.

The tests will include the following:

- Questions about your medical history, including questions about your emotional and psychiatric history. This may include any previous medical or psychiatric problems or treatment and may include questions about difficult experiences you may have experienced.

- A physical examination and vital signs that will include measures of your blood pressure, pulse, temperature, and body weight.

- An ECG to assess for cardiovascular conditions.

- A sample of your blood (14mL) for routine laboratory testing, including a pregnancy test, and other hematology and biochemistry tests. Pregnancy test must be negative to continue in the study.

- A urine test for drugs of abuse, including stimulants, sedatives, opioids, and cannabis. We will not report findings of drugs to any authorities.

- Study Questionnaires

During the study you will complete a set of questionnaires four (4) times. The questions will be about your emotional well-being, mental health symptoms, and your quality of life The questionnaire package will take approximately 30-45 minutes to complete. You can complete the questionnaires at home using paper-and-pencil or on our online system. You may also choose to complete the questionnaires over the phone or at the hospital with a research assistant who can read questions aloud, and you can tell them your answers.

***Psychotherapy and Study Drug sessions:***

If you are found eligible to continue in the study, we will schedule your first psychotherapy session with the therapy team.

- **Initial three (3) psychotherapy sessions** (about 75 minutes each):

These sessions will be spaced about one week apart. You will meet with your primary therapist and could be in person, by telephone, or through virtual care.

During each session, you will talk about your cancer symptoms and management, your medical history, the ways cancer has affected your life and relations with close others, what you would like to achieve during the study, and preparing for the future.

You may choose to bring a loved one/caregiver to one or more of these sessions to help prepare them for the psychedelic session (when you will take psilocybin), answer any questions, and help them understand how to support you after the session.

- **Preparatory session** (about 90 minutes):

This session will occur in-person with your two therapists. This session will take place within one week before you receive psilocybin. This session will include detailed education about what you can expect when you receive psilocybin.

- **Psilocybin/Study Drug session:**

  - ✓ Do NOT take any herbal supplements, non-prescription medications, or prescription medications that have not been discussed and approved with the study team for 1 week before the psilocybin session

  - ✓ Do NOT use any psychoactive drug (including alcoholic beverages) 24 hours of your psilocybin session

  - ✓ Do NOT take or use nicotine for 2 hours before and 7 hours after you take psilocybin

  - ✓ Do NOT change the amount of caffeine (e.g., coffee, tea) that you usually take in the morning before your psilocybin session

  - ✓ Do NOT take any medications on the morning of your psilocybin session (with the exception of daily and as needed opioid pain medication)

You may need to stop some medications prior to receiving psilocybin. You should *only* stop medications if the study doctor gives you specific instructions to do so. If you are eligible to receive the study drug, you will then receive one dose of the study drug, psilocybin.

This session will be an average of 6-8 hours in length following ingestion of a 25mg capsule of psilocybin. This session will occur in the treatment setting with two therapists present.

Before the psilocybin session:

- If you can become pregnant, you will take a urine pregnancy test. If you are pregnant, you cannot participate in this study.

Then the psilocybin session will begin. The therapists will help you get into a comfortable position. Relaxation exercises, including breathing and mindfulness, will be used to help ease any anxiety you may have.

You will then receive a 25mg capsule of psilocybin. After taking the medicine, you will sit or lie down in a comfortable position. You will be provided with eyeshades that you may use. You will listen to music for much of the session, either through headphones or speakers. Lying or sitting in a comfortable position and listening to music are meant to evoke thoughts and feelings.

The two therapists will remain with you, and they will help you if you need them to. They will check in with you and ask you to talk to them at least once an hour, but you can talk to them whenever you wish. You may ask for support from the therapists whenever you wish. There may be times when the therapy team will suggest that you stop talking for a while in order to pay attention to your thoughts and feelings.

During the preparatory session, your therapist will discuss and see if you agree to the use of therapeutic touch. Therapeutic touch refers to hand holding, or the therapist placing a hand on your shoulder, upper back, arm, lower leg or foot. You may request or your therapist may offer therapeutic touch during the psilocybin session. *This is optional.* You do not need to agree to therapeutic touch in order to participate in the study. You may also decline therapeutic touch at any time during the study, including during the psilocybin session.

There will be beverages available (water, juice, sports drink) and you will be encouraged to drink fluids. In the afternoon, a light snack will also be provided.

The therapy team will watch for any side effects (unwanted effects or health problems) and monitor your blood pressure, temperature, and pulse.

- ✓ Tell your therapy team, if you have any symptoms including confusion, light-headedness, dizziness, chest pain, or shortness of breath. You will receive treatment if needed.

- ✓ You must have someone drive you after the session to where you are staying (home, hotel or another location), because psilocybin may affect your alertness and concentration on the evening of the dosing session.

- ✓ Do NOT drive for a day after you took psilocybin.

- **Integration and follow up sessions:**

The integration and follow-up sessions are to help you express, understand, and connect any thoughts or feelings you may be having about your cancer, and to talk about your experience during the psilocybin session.

- ✓ **Integration session** (about 90 minutes):

This session will occur the day after the psilocybin-dosing session in person at the hospital. A family caregiver will be invited to attend a portion of this session.

With the support of both therapists, this session will involve reflection on the perspectives, insights, and experiences of the psilocybin session; understanding these in the context of your individual psychology; and considering their impact on your orientation to the future, hope, and mortality. Some attention will be paid to helping you develop practices to continue your integration process at home.

- ✓ **Follow-up session(s)** (about 90 minutes each):

These two sessions with the primary therapist will take place within two weeks of the integration session. They may be in person, by telephone, or through virtual care.

These sessions will continue to explore and integrate the PEARL therapy experience. For example, discussions may involve disease and symptom management; changes in personal relationships, spiritual well-being, or maintaining a sense of meaning and purpose; and preparing for the future, sustaining hope, and facing mortality.

- ✓ **One month after your final follow-up session**

You will be asked to participate in an interview. You will be asked about your experiences with PEARL therapy, psilocybin, and any positive or negative effects. The interview will last approximately 60 minutes and can take place in-person, via telephone, or via secure video call. The interview will be recorded so that the study team will have accurate records.

**Risks:**

Taking part in this study has risks. Some of these risks we know about. There is also a possibility of risks that we do not know about. Please contact the study doctor if you have any side effects even if you do not think it has anything to do with this study.

***Psilocybin***

Common side effects 20 to 49%

- Mild to moderate increase in heart rate and blood pressure during the dosing session
- Headache – typically on the day of, or the day following, the dosing session

Less Common (1 to 19%)

- Nausea – typically experienced during the dosing session

- Transient psychological distress (e.g., anxiety, or paranoia) during the dosing session (2%)
- Higher anxiety and/or rapid emotional changes/intense emotions in the days or weeks following the psilocybin session (1%)

Rare (less than 0.1%)

- Psychosis (for example, hearing voices that are not really there, or feeling paranoid)

In recent studies of psilocybin-assisted therapy among people with cancer, no instances of psychosis have been reported. In other clinical studies of different groups of people, the risk of psychosis appears to be very low.

There have been no human case reports or studies involving the effects of psilocybin on pregnancy. It is recommended that people who are pregnant avoid using psilocybin. You must not become pregnant, father a baby, or donate sperm while receiving psilocybin and for 1 month afterwards. Your study therapist will discuss methods with you to ensure that you do not become pregnant or father a baby during the study. If you do become pregnant or father a baby during the study you should immediately notify your study therapist.

The manufacturer of the investigational product, Usona Institute, would like your permission to follow your pregnancy to gather information on the outcome of your pregnancy and/or the health of the baby. Should pregnancy occur, and you agree to be followed, you will be asked to sign a separate consent form.

***Psychotherapy and interviews:***
You may feel upset or increased symptoms at the review of your emotional experiences or past traumas, or you may feel fatigue.

***Blood Draw:***
You may experience pain, bleeding, and/or bruising, swelling, and rarely infection and temporary redness of the skin at the place where the needle is put into your arm. You may experience light-headedness.

**Benefits:**

You may or may not receive direct benefit from being in this study.

Information obtained from this study may help doctors and researchers to improve treatment for advanced cancer patients and inform therapy approaches. This may benefit other patients in the future.

**Reminders and Responsibilities:**

- Provide the study team the name and contact information (telephone number, cell phone number) of a relative, spouse or close friend. This contact is necessary in case of medical emergency, should you become at risk of hurting yourself or

someone else, or if the study team cannot get in touch with you, so they can reach that person to let them know what is going on or to find out if you are okay.

- If you are in distress during this study and require support:
  - During office hours, please call the study team.
  - In an emergency, go to your nearest hospital emergency room and then notify the study team.

- Inform the study doctors if any of the following things happen:
  - you have an increase in symptoms from medications you have taken in the past
  - you have a new symptom or medical issue
  - you need to contact your outside therapist other than for the usual appointments
  - you start or stop taking a prescription or an over-the-counter medication that you have not previously cleared with the study team
  - you go to the hospital for any reason

## Alternatives to Being in the Study:

You do not have to join this study to receive treatment for your condition. If you decline being in this study, your care will not be affected.  You may receive other treatments that may help resolve your symptoms psychological or emotional distress.

There are approved medicines and other psychotherapies that may help with anxiety, depression, or other cancer-related distress.

If you are currently undergoing psychotherapy and/or taking medicine, you could continue with those for a longer period of time. The therapy team can discuss the alternatives and their potential risks and benefits with you.

## Confidentiality:

Your data will be shared as described in this consent form or as required by law. All personal information such as your name, address, phone number, OHIP number, and family physician's name will be removed from the data and will be replaced with a number and your initials. A list linking the number with your name will be kept by the study doctor in a secure place, separate from your file.

### Personal Health Information

If you agree to join this study, the study doctor and their study team will look at your personal health information and collect only the information they need for the study. Personal health information is any information that could identify you and may include:

- name
- address

- month and year of birth
- race and ethnicity
- new or existing medical records, that includes types, dates and results of medical tests or procedures

The following people may come to the hospital or be given remote access to an electronic portal (via the internet) to look at the study records, including video recordings, and your personal health information to check that the information collected for the study is correct and to make sure the study is following proper laws and guidelines:

- Representatives of the University Health Network (UHN) including the UHN Research Ethics Board
- Representatives of Health Canada or other regulatory bodies (groups of people who oversee research studies) outside of Canada, such as the United States Food and Drug Administration.

These individuals have completed privacy training and signed confidentiality agreements and/or are required by law to keep your information confidential.

If you experience a severe reaction to the study drug, we may share information on the reaction and its symptoms as well as your month and year of birth, sex, and country with the manufacturer of the investigational product, Usona Institute, based in Madison, Wisconsin, in the United States. This information will be identified by the study number.

Whether on-site or remotely, UHN makes all efforts to ensure that your information is shared in a way that is secure and private (encrypted). However, any electronic communication carries some risk of third parties gaining unauthorized access to information.

All information collected during this study, including your personal health information, will be kept confidential.

<u>Research Information in Shared Clinical Records</u>

If you participate in this study, information about you from this research project may be stored in your hospital file and in the UHN computer system. The UHN shares the patient information stored on its computers with other hospitals and health care providers in Ontario so they can access the information if it is needed for your clinical care. The study team can tell you what information about you will be stored electronically and may be shared outside of the UHN. If you have any concerns about this, or have any questions, please contact the UHN Privacy Office at 416-340-4800, x6937 (or by email at privacy@uhn.ca).

<u>Study Information that Does Not Identify You</u>

Any information about you that may be sent out of the hospital will have your participant code only, and will not show your name or address, or any information that directly identifies you.

You will not be named in any reports, publications, or presentations.

*<u>Storage of Treatment Session Recordings:</u>*

Recordings of the treatment sessions will be stored securely *<u>for 15 years as required</u>*.

The recordings will be saved in a secure UHN cloud-based OneDrive folder or on a secure device in a locked cabinet only accessible to study team members.

*<u>Storage of Qualitative Interview Recordings:</u>*

The recordings will be stored in a secure UHN cloud-based OneDrive folder only accessible to study team members and as required by regulations. The recordings will be transcribed, verified, and analyzed. One year after study completion, the recordings will be deleted.

The de-identified transcripts will be saved in the participant study record in the UHN SharePoint for 15 years and could be re-analyzed for the study purposes during these 15 years.

**<u>Voluntary Participation:</u>**

Your participation in this study is voluntary. You may decide not to be in this study, or to be in the study now, and then change your mind later. You may leave the study at any time without affecting your care.

We will give you any new information that is learned during the study that might affect your decision to stay in the study.

**<u>Withdrawal from the Study:</u>**

You may withdraw from the study at any time. If your physician feels that taking part in the study is causing harm, they may also remove you from the study. We will work with you to help you access alternative treatments should this happen.

If you decide to leave the study, the information about you that was collected before you left the study will still be used in order to help answer the research questions. No new information will be collected without your permission.

## Costs and Reimbursement:

You will not be paid for taking part in this study. You will not have to pay for any of the procedures involved with this study. You will not be reimbursed for any transportation expenses.

## Rights as a Participant:

If you are harmed as a direct result of taking part in this study, all necessary medical treatment will be made available to you at no cost.

By signing this form, you do not give up any of your legal rights against the investigators, sponsor or involved institutions for compensation, nor does this form relieve the investigators, sponsor or involved institutions of their legal and professional responsibilities.

## Conflict of Interest:

Researchers have an interest in completing this study. Their interests should not influence your decision to participate in this study.

## Data Safety Monitoring:

A Data Safety Monitoring Board is a group of experts who will be reviewing the data throughout this research study to see if there are unexpected or more serious side effects than described in this form. The experts in the group are employees of the hospital/clinic.

## Study Registration and Results:

A description of this clinical trial will be available on http://www.ClinicalTrials.gov. This website will not include information that can identify you. At most, the website will include a summary of the results. You can search this website at any time.

## Questions about the Study:

If you have any questions, concerns or would like to speak to the study team for any reason, please email sarah.hales@uhn.ca or call 416-946-4501 x2551.
(Please do NOT use email to discuss information you think is sensitive. Do NOT use email in an emergency since email may be delayed.)
If you have any questions about your rights as a research participant or have concerns about this study, call the Chair of the University Health Network Research Ethics Board (UHN REB) or the Research Ethics office number at 416-581-7849. The REB is a group of people who oversee the ethical conduct of research studies. The UHN REB is not part of the study team. Everything that you discuss will be kept confidential.

**You will be given a signed copy of this consent form.**

**Consent:**

This study has been explained to me and any questions I had have been answered. I know that I may leave the study at any time. I agree to the use of my information as described in this form. I agree to take part in this study.

| | | |
|---|---|---|
| Print Study Participant's Name | Signature of Participant | Date |

*For person obtaining consent:* My signature means that I have explained the study to the participant named above. I have answered all questions.

| | | |
|---|---|---|
| Print Name of Person Obtaining Consent | Signature of Person Obtaining Consent | Date |
